# Supplementary material for: End-to-end differentiable construction of molecular mechanics force fields
Source: Chem Sci. 2022 Sep 8;13(41):12016–33. doi: 10.1039/d2sc02739a (PMC9600499; doi:10.1039/d2sc02739a)
Supplement: SC-013-D2SC02739A-s001 [file SC-013-D2SC02739A-s001.pdf]

Electronic Supplementary Material (ESI) for Chemical Science.
This journal is © The Royal Society of Chemistry 2022

Supplementary Information: End-to-End differentiable construction of molecular mechanics force fields

Yuanqing Wang (ORCID: 0000-0003-4403-2015)[1, 3, 6], **Josh Fass (ORCID: 0000-0003-3719-266X)**[1, 2‡], **Benjamin Kaminow (ORCID: 0000-0002-2266-3353)**[1, 2], **John E. Herr (ORCID: 0000-0001-5639-1520)**[1], **Dominic Rufa (ORCID: 0000-0003-0930-9445)**[1, 4], **Ivy Zhang (ORCID: 0000-0003-0628-6276)**[1, 2], **Iván Pulido (ORCID: 0000-0002-7178-8136)**[1], **Mike Henry (ORCID: 0000-0002-3870-9993)**[1], **Hannah E. Bruce MacDonald (ORCID: 0000-0002-5562-6866)**[1§], **Kenichiro Takaba (ORCID: 0000-0002-2481-8830)**[1, 5], **John D. Chodera (ORCID: 0000-0003-0542-119X)**[1]

[1]Computational and Systems Biology Program, Sloan Kettering Institute, Memorial Sloan Kettering Cancer Center, New York, NY 10065; [2]Tri-Institutional PhD Program in Computational Biology and Medicine, Weill Cornell Medical College, Cornell University, New York, NY 10065; [3]Physiology, Biophysics, and System Biology Ph.D. Program, Weill Cornell Medical College, Cornell University, New York, NY 10065; [4]Tri-Institutional PhD Program in Chemical Biology, Weill Cornell Medical College, Cornell University, New York, NY 10065; [5]Pharmaceutical Research Center, Advanced Drug Discovery, Asahi Kasei Pharma Corporation, Shizuoka 410-2321, Japan; [6]MFA Program in Creative Writing, Division of Humanities and Arts, City College of New York, City University of New York, New York, NY 10031

***For correspondence:**
yuanqing.wang@choderalab.org (YW); john.chodera@choderalab.org (JDC)

Present address: ‡Relay Therapeutics, Cambridge, MA 02139; §MSD Ltd., Francis Crick Institute, Kings Cross, London, NW1 1AT, UK

A Detailed Methods

A.1 Code and Parameter Availability

The Python code used to produce the results discussed in this paper is distributed open source under MIT license [`https://github.com/choderalab/espaloma`]. Core dependencies include PyTorch 1.9.1 [1], Deep Graph Library 0.6.0 [2], the Open Force Field Toolkit 0.10.0 [3, 4], and OpenMM 7.7.0 [5].

Describe how espaloma can be used in OpenMM via openmmtools, and describe which model is available as espaloma-0.0.2

A.2 Datasets

The typed ZINC validation subset distributed with parm@Frosst [6] was used in atom typing classification experiments (Section 1.1).

For MM fitting experiments (Section 2), we employed molecules the PhAlkEthOH dataset [7], parametrized with GAFF-1.81 [8] using Antechamber [9, 10] from AmberTools21, and generated molecular dynamics (MD) snapshots with annotated energies according to the procedure detailed below (Section A.4). We filtered out molecules with a gap between minimum and maximum energy larger than 0.1 Hartree (62.5 kcal/mol).

For QM fitting experiments (Section 3), datasets hosted on QCArchive [11] are used. We filter out snapshots with energies more than 0.1 Hartree (62.5 kcal/mol) higher than the minima. Within all datasets, we randomly select training, test, and validation sets with 80:10:10 partitions.

A.3 Machine learning experimental details

The input features of the atoms included the one-hot encoded element, as well as the hybridization, aromaticity, (various sized-) ring membership, and formal charge thereof, assigned using the OpenEye Toolkit (OpenEye Scientific Software).

All models are trained with 5000 epochs with the Adam optimizer [12]; early stopping was used to select the epoch with lowest validation set loss.

Hyperparameters, namely choices of graph neural network layer architectures (GIN [13], GCN [14], GraphSAGE [15], SGConv [16]), depth of graph neural network and of Janossy pooling network (3, 4, 5, 6), activation functions (ReLU, sigmoid, tanh), learning rates (1e-3, 1e-4, 1e-5), and per-layer units (16, 32, 64, 128, 256, 512) were briefly optimized with a grid search using validation sets on the MM fitting experiment. As a result, we use three 128-units GraphSAGE [15] layers with ReLU activation function for stage I and four 128-units feed-forward layers with ReLU activation for stage II and III. Reported metrics: R^2: the coefficient of determination, RMSE: root mean square error, MAPE: mean absolute percentage error; note that the MAPE results we report is not multiplied by 100, and therefore denotes the fractional error. The annotated 95% confidence intervals are calculated by bootstrapping the test set 1000 times to account for finite-size effects in the composition of the test set.

A.4 Molecular dynamics simulation details

High-temperature MD simulations described in Section 2 were initialized using RDKit's default conformer generator followed by energy minimization in OpenMM 7.5, with initial velocities assigned randomly to the target temperature. Vacuum trajectories were simulated without constraints using `LangevinIntegrator` from OpenMM [5] using a temperature of 500 K, collision rate of 1/picosecond, and a timestep of 1 fs. 500 samples (5 ns) are collected with 10000 steps (10 ps) between each sample.

A.5 Alchemical free energy calculations

We used the perses 0.9.5 relative alchemical free energy calculation infrastructure [17] (based on OpenMM 7.7 [5] and openmmtools 0.21.2 [18]) to compare performance on the Tyk2 kinase:inhibitor benchmark set from the Schrodinger JACS benchmark set [19] as curated by the OpenFF protein-ligand benchmark 0.2.0 [20]. In order to assess the impact of espaloma small molecule parameters and charges in isolation, we used the Amber ff14SB protein force field [21], and performed simulations with either OpenFF 1.2.0 (`openff-1.2.0`) or the espaloma Joint model trained on OpenFF Gen2 Optimization and PepConf datasets (`espaloma-0.2.2`) available through the openmmforcefields 0.11.0 package [22]. Notably, none of the ligands appearing in this set appear in the training set for either force field. All systems were explicitly solvated with a 9 Åbuffer around the protein with TIP3P water [23] and use the Joung and Cheatham monovalent counterion parameters [24] to model a neutral system with 300 mM NaCl salt. The same transformation network provided in the OpenFF protein-ligand benchmark set was used to compute alchemical transformations, and absolute free energies up to an additive constant were estimated from a least-squares estimation strategy [25] as implemented in the OpenFF arsenic package [26]. Both experimental and calculated absolute free energies were shifted to their respective means before computing statistics, as in [19].

Alchemical free energy calculations used replica exchange among Hamiltonians with Gibbs sampling complete mixing exchanges each iteration [27], simulating 5 ns/replica with 1 ps between exchange attempts. 12 alchemical states were used. Simulations were conducted at 300 K and 1 atm using a Monte Carlo Barostat and Langevin BAOAB integrator [28] with bonds to hydrogen constrained, a collision rate of 1/ps, 4 fs timestep, and heavy hydrogen masses. Atom mappings were generated from the provided geometries in the benchmark set, mapping atoms that were within 0.2Å and subsequently correcting the maps to be valid with the `use_given_geometries` functionality of perses.

B Espaloma can generate fast and accurate partial charges and valence parameters simultaneously

We integrated the charge equilibration approach into an espaloma model where parameters of the charge equilibration model $\{e_i, s_i\}$ and the bonded (bond, angle, and torsion) parameters are optimized jointly. The resulting model is trained by augmenting the loss function to include a term that penalizes the deviation from AM1-BCC partial charges for the molecules in the training set. On the one hand, one can calculate the Coulomb energy term using this predicted set of charges and incorporate this directly into the energy MSE

PhAlkEthOH experiment (combination of independent models)		energy RMSE (kcal/mol)		charge RMSE (e)	
valence force field	charge model	Train	Test	Train	Test
openff-1.2.0	AM1-BCC	$1.6071_{1.5197}^{1.6915}$		reference	
openff-1.2.0	espaloma	$1.6286_{1.5861}^{1.6628}$	$1.7072_{1.6361}^{1.7913}$	$0.0072_{0.0071}^{0.0073}$	$0.0072_{0.0070}^{0.0073}$
espaloma	AM1-BCC	$0.8656_{0.8225}^{0.9131}$	$1.1398_{1.0715}^{1.2332}$	reference	
espaloma	espaloma	$0.9596_{0.9100}^{1.0101}$	$1.2216_{1.1529}^{1.2766}$	$0.0072_{0.0071}^{0.0073}$	$0.0072_{0.0070}^{0.0073}$
Joint Model, Loss=	$\text{MSE}(U_{\text{qm}}(\mathbf{x}), \hat{U}_{\texttt{valence}}(\mathbf{x}; \Phi_{\text{NN}}) + \hat{U}_{\texttt{charge}}(\mathbf{x}; \Phi_{\text{NN}}) + U_{\text{LJ}}(\mathbf{x}))$	$0.8646_{0.8186}^{0.9180}$	$1.0839_{1.0462}^{1.1228}$	$0.3218_{0.3173}^{0.3258}$	$0.3230_{0.3184}^{0.3290}$
	$\text{MSE}(U_{\text{qm}}(\mathbf{x}), \hat{U}_{\texttt{valence}}(\mathbf{x}; \Phi_{\text{NN}}) + U_{\texttt{AM1-BCC}}(\mathbf{x}) + U_{\text{LJ}}(\mathbf{x})) + \text{MSE}(q, \hat{q}(\boldsymbol{\Phi}_{\textbf{NN}}))$	$0.8336_{0.7882}^{0.8877}$	$1.0987_{1.0318}^{1.1697}$	$0.0075_{0.0073}^{0.0077}$	$0.0076_{0.0075}^{0.0077}$
	$\text{MSE}(U_{\text{qm}}(\mathbf{x}), \hat{U}_{\texttt{valence}}(\mathbf{x}; \Phi_{\text{NN}}) + \hat{U}_{\texttt{charge}}(\mathbf{x}; \Phi_{\text{NN}}) + U_{\text{LJ}}(\mathbf{x})) + \text{MSE}(q, \hat{q}(\boldsymbol{\Phi}_{\textbf{NN}}))$	$0.9912_{0.9531}^{1.0443}$	$1.2921_{1.2403}^{1.3323}$	$0.0138_{0.0136}^{0.0140}$	$0.0137_{0.0135}^{0.0139}$

Table S1. Espaloma can jointly predict partial charges through a simple charge-equilibration model that learns electronegativity and hardness parameters. Using the OpenFF PhAlkEthOH dataset (244,036 snapshots over 7,408 molecules containing only carbon, oxygen, and hydrogen atoms), we compare the performance of indepdently trained and jointly trained espaloma models in reproducing snapshot potential energies and AM1-BCC partial charges. In the first two rows, we train two independent models to predict the energy of conformations and charges of atoms; in the third row, we use a joint model where the latent embedding is shared between these two tasks. As a reference, the RMSE between AM1-BCC charges assigned by two different chemoinformatics toolkits—Ambertools 21 [9] and OpenEye Toolkit—are $0.0126_{0.0124}^{0.0129}$ (e).

loss function (shown in the first row in the second half of Table S1). This approach, although it maintains a relatively accurate energy prediction, leads to a large charge RMSE, since no reference charge is provided. On the other hand, we can penalize the derivation from the reference charges by adding an MSE loss on the charges with a tunable weight as a hyperparameter (second row), which we tune on the validation set to be $1e-3$. This setting results in satisfactory performance in both energy and charge prediction. Finally, if we combine both losses (third row), we observe worse performance on test set energy predictions, which could be attributed to the repeated strong regularization on charge parameters.

C A graph theoretic view of Class I molecular mechanics force fields

Consider a molecular graph $\mathcal{G}$ where atoms map to vertices $\mathcal{V}$ and covalent bonds map to edges $\mathcal{E}$. In a class I molecular mechanics force field [29–35], the parameters Φ_{FF} assigned to a molecule graph $\mathcal{G}$ define how the total potential energy of a conformation $\mathbf{x} \in \mathbb{R}^{|\mathcal{G}|*3}$ is computed from independent bond, angle, torsion, and nonbonded energy terms given the complete set of molecular mechanics parameters Φ_{FF}.

$$
\begin{aligned}
U_{\text{MM}}(\mathbf{x}; \mathcal{G}, \Phi_{\text{FF}}) \quad &= \sum_{(v_i, v_j) \in \mathcal{G}_{\text{bond}}} U_{\text{bond}} \left(r(\mathbf{x}; v_i, v_j); k_r(\Phi_{\text{FF}}; v_i, v_j), r_0(\Phi_{\text{FF}}; v_i, v_j) \right) \\
&+ \sum_{(v_i, v_j, v_k) \in \mathcal{G}_{\text{angle}}} U_{\text{angle}} \left(\theta(\mathbf{x}; v_i, v_j, v_k); K_\theta(\Phi_{\text{FF}}; v_i, v_j, v_k), \theta_0(\Phi_{\text{FF}}; v_i, v_j, v_k) \right) \\
&+ \sum_{(v_i, v_j, v_k, v_l) \in \mathcal{G}_{\text{torsion}}} U_{\text{torsion}} \left(\phi(\mathbf{x}; v_i, v_j, v_k, v_l); \{ K_{\phi,n}(\Phi_{\text{FF}}; v_i, v_j, v_k, v_l) \}_{n=1}^{n_{\text{max}}}, \phi_0(\Phi_{\text{FF}}; v_i, v_j, v_k, v_l) \right) \\
&+ \sum_{(v_i, v_j) \in \mathcal{G}_{\text{Coulomb}}} U_{\text{Coulomb}} \left(r(\mathbf{x}; v_i, v_j); q(\Phi_{\text{FF}}; v_i), q(\Phi_{\text{FF}}; v_j) \right) \\
&+ \sum_{(v_i, v_j) \in \mathcal{G}_{\text{van der Waals}}} U_{\text{van der Waals}} \left(r(\mathbf{x}; v_i, v_j); \sigma(\Phi_{\text{FF}}; v_i, v_j), \epsilon(\Phi_{\text{FF}}; v_i, v_j) \right)
\end{aligned}
\tag{1}
$$

Here, the sets $\mathcal{G}_{\text{bond}}, \mathcal{G}_{\text{angle}}, \mathcal{G}_{\text{torsion}}$ denote the duples, triples, and quadruples of bonded atoms (vertices) in $\mathcal{G}$, while $\mathcal{G}_{\text{Coulomb}}$ and $\mathcal{G}_{\text{van der Waals}}$ denotes the set of atom (vertex) pairs separated by at least *three* edges, since interactions separated by fewer edges are generally excluded. ϵ_0 denotes the vacuum electric permittivity. The potential terms depend on distances $r(\mathbf{x}; v_i, v_j)$, angles $\theta(\mathbf{x}; v_i, v_j, v_k)$, and torsions (dihedral angles) $\phi(\mathbf{x}; v_i, v_j, v_k, v_l)$ measured for the corresponding atoms from the positions vector $\mathbf{x}$. The various parameter functions—bond force constant k_r and equilibrium distance r_0, angle force constant K_θ and equilibrium angle θ_0, periodic torsion term barrier height $K_{\phi,n}$ and phase ϕ_0 for periodicity n, partial charge q, Lennard-Jones radius σ and well depth ϵ—extract the parameters corresponding to specific sets of atoms (vertices) from the vector of molecular mechanics parameters Φ_{FF} to compute that contribution to the total potential energy.

The individual potential energy terms are computed by functions that generally take simple harmonic or periodic forms with respect to bond lengths and angles in terms of the parameters for those specific

interactions:

$$U_{\text{bond}}(r; k_r, r_0) = \frac{K_r}{2}(r - r_0)^2 \tag{2}$$

$$U_{\text{angle}}(\theta; K_\theta, \theta_0) = \frac{K_\theta}{2}(\theta - \theta_0)^2 \tag{3}$$

$$U_{\text{torsion}}(\phi; \{K_{\phi,n}\}, \phi_0) = \sum_{n=1}^{n_{\max}} K_{\phi,n}\left[1 + \cos(n\phi)\right] \tag{4}$$

$$U_{\text{Coulomb}}(r; q_i, q_j) = \frac{1}{4\pi\epsilon_0}\frac{q_i\, q_j}{r} \tag{5}$$

$$U_{\text{van der Waals}}(r; \sigma, \epsilon) = 4\epsilon\left[\left(\frac{\sigma}{r}\right)^{12} - \left(\frac{\sigma}{r}\right)^{6}\right] \tag{6}$$

where k_r and k_θ denote force constants for bonds and angles, r_0 and θ_0 denote equilibrium bond lengths and angles, $K_{\phi,n}$ denotes a torsion energy factor (which can be positive or negative) for periodicity n, ϵ_0 is the permittivity of free space, and q_i denote partial charges. For force fields like AMBER [21] and CHARMM [36], the effective Lennard-Jones well depth ϵ_{v_i,v_j} and radius σ_{v_i,v_j} parameters for an interacting pair of atoms are computed from atomic parameters for atoms v_i and v_j using Lorentz-Berthelot combining rules [37],

$$\sigma(\Phi_{\text{FF}}; v_i, v_j) = \frac{1}{2}(\sigma_{v_i} + \sigma_{v_j}) \tag{7}$$

$$\epsilon(\Phi_{\text{FF}}; v_i, v_j) = \sqrt{\epsilon_{v_i}\epsilon_{v_j}} \tag{8}$$

though alternative combining rules [38] or even pair-specific parameters [39] are also possible.

Typically, atom type based force field parameterization engines [40] such as those used in AMBER [21] or CHARMM [36] assign parameters based on templates (for biopolymer residues or solvents) or through chemical perception algorithms (as in the case of GAFF [9, 10] or CGenFF [41, 42]). More recently, an approach to assigning atom, bond, angle, and torsion parameters directly based on the common SMARTS/SMIRKS chemical perception language was introduced, which bypasses the need to define parameter classes in terms of atom types, but still retains discrete interaction types [40]. Automatically fitting these type definitions still represents an intractable mixed discrete-continuous optimization problem. In the next section, we will show how a continuous, differentiable model can assign parameters directly, without the use of discrete atom or interaction types.

D A brief introduction to graph neural networks

In the context of molecular machine learning, molecules are modelled as undirected graphs of bonded atoms, where each atom and bond can carry attributes reflecting their chemical nature from which complex chemical features can be learned. If we write this as a tuple of three sets,

$$\mathcal{G} = \{\mathcal{V}, \mathcal{E}, \mathcal{U}\} \tag{9}$$

Here, $\mathcal{V}$ is the set of the vertices (or nodes) (atoms), $\mathcal{E}$ the set of edges (bonds), and $\mathcal{U} = \{\mathbf{u}\}$ the universal (global) attribute.

In a graph neural network (GN) a set of functions (with learnable parameters Φ_{NN}) govern the three stages used in both training and inference of a graph neural network: *initialization*, *propagation*, and *readout*. Following one of the most general description of the message-passing procedure in the propagation stage in [43], we briefly review the message-passing steps in graph neural networks, where node attribuves $\mathbf{v}$, edge attributes $\mathbf{e}$, and global attributes $\mathbf{u}$ are updated according to:

$$\mathbf{e}_k^{(t+1)} = \phi^e(\mathbf{e}_k^{(t)}, \sum_{i\in\mathcal{N}_k^e} \mathbf{v}_i, \mathbf{u}^{(t)}), \quad \text{edge update} \tag{10}$$

$$\bar{\mathbf{e}}_i^{(t+1)} = \rho^{e\to v}(E_i^{(t+1)}), \quad \text{edge-to-node aggregate} \tag{11}$$

$$\mathbf{v}_i^{(t+1)} = \phi^v(\bar{\mathbf{e}}_i^{(t+1)}, \mathbf{v}_i^{(t)}, \mathbf{u}^{(t)}), \quad \text{node update} \tag{12}$$

$$\bar{\mathbf{e}}^{(t+1)} = \rho^{e\to u}(E^{(t+1)}), \quad \text{edge-to-global aggregate} \tag{13}$$

$$\bar{\mathbf{u}}^{(t+1)} = \rho^{v\to u}(V^{(t)}), \quad \text{node-to-global aggregate} \tag{14}$$

$$\mathbf{u}^{(t+1)} = \phi^u(\bar{\mathbf{e}}^{(t+1)}, \bar{\mathbf{v}}^{(t+1)}, \mathbf{u}^{(t)}), \quad \text{global update} \tag{15}$$

where $E_i = \{\mathbf{e}_k, k \in \mathcal{N}_i^v\}$ is the set of attributes of edges connected to a specific node, $E_i = \{e_k, k \in 1, 2, ..., N^e\}$ is the set of attributes of all edges, V is the set of attributes of all nodes, and $\mathcal{N}^v$ and $\mathcal{N}^e$ denote the set of indices of entities connected to a certain node or a certain edge, respectively. ϕ^e, ϕ^v, and ϕ^u are update functions that take the *environment* of the an entity as input and update the attribute of the entity, which could be stateful or not; $\rho^{e\rightarrow v}$, $\rho^{e\rightarrow u}$, and $\rho^{v\rightarrow u}$ are aggregation functions that aggregate the attributes of multiple entities into an *aggregated* attribute which shares the same dimension with each entity. Note that it is common that the edges do not hold attribute but only pass messages onto neighboring nodes. For all models we survey here, edge-to-global update does not apply and global attribute does not present until the readout stage, when a sum function is applied to form the global representation ($\mathbf{u} = \sum_i \mathbf{v}_i$). We review the specifics of the graph neural network architectures that we considered here in the ESI (Table S2).

D.1 Training and inference

While traditional force fields based on discrete atom types are only differentiable with respect to the molecular mechanics parameters they assign, our model is fully differentiable in *all* model parameters Φ_{NN} that govern both the assignment of continuous atom embeddings h_v (which replace discrete atom types) and subsequent assignment of MM parameters Φ_{FF}. We can therefore use gradient-based optimization to tune all of these parameters to fit arbitrary differentiable target functions, such as energies and forces of snapshots of molecules, equilibrium physical property measurements, fluctuation properties, or experimentally measured free energy differences. In this way, straightforward gradient-based optimization simultaneously learns the continuous equivalent of atom typing simultaneous with parameter assignment strategies, effectively solving the intractable traditional force field parmaeterization problem of mixed continuous-discrete optimization by replacing it with a fully continuous optimization problem.

D.1.1 A linear basis facilitates bond and angle parameter optimization

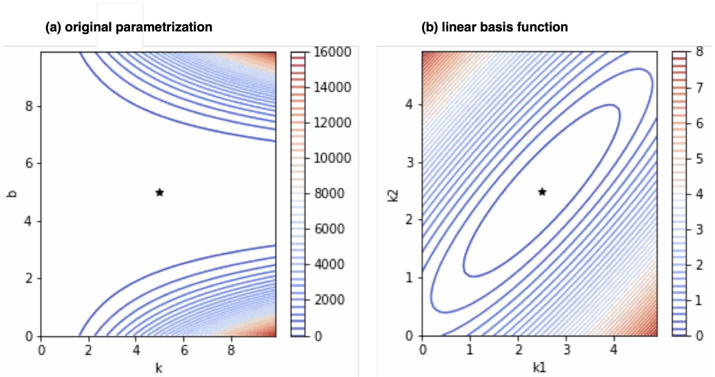

Figure S1. Linear basis parameterization of bonds and angles facilitates robust optimization. A one-dimensional harmonic oscillator system ($u = \frac{K_0}{2}(x - b_0)^2$) was simulated with $K_0 = 5$, $b_0 = 5$ as reference parameters. Training set conformations x were sampled from a uniform distribution with $x \sim \mathrm{Uniform}(4, 6)$. The loss function (squared energy error) as a function of original MM parameters (K, b) shown in (a) is clearly more difficult to optimize due to the large difference in gradient magnitudes between K and b parameters, while the linear basis parametrization (Section D.1.1) showing the loss as a function of transformed (K_1, K_2) in (b) is much simpler to optimize.

The harmonic functional form (Equation 6),

$$u(x; K, b) = \frac{K}{2}(x - b)^2, \tag{16}$$

is frequently used in MM potentials for modeling bond and angle energies. Although one can directly optimize force constants K and equilibrium bond or angle values b, this empirically result in significant difficulties in training (Figure S1). This can be seen through the loss function and its gradient for a simple toy model

consisting of a single bond and reference parameters (K_0, b_0):

$$L(K,b) = \sum_{n=1}^{N}[u(x_n;K,b) - u(x_n;K_0,b_0)]^2 \quad (17)$$

$$= \sum_{n=1}^{N}[\frac{K}{2}(x_n - b)^2 - \frac{K_0}{2}(x_n - b_0)^2]^2 \quad (18)$$

Qualitatively, the loss function landscape for K around K_0 is relatively flat, while it is steeply varying for b around b_0, frustrating efficient optimization; Figure S1a demonstrates this for a simple toy system. To circum-vent this issue, we use the approach described by Vanommeslaeghe et al. [44] and translate the harmonic functional form Equation 16 in which (K, b_0) are optimized instead into a linear combination (K_1, K_2) of basis functions is optimized instead:

$$u(x;K_1,K_2) = \underbrace{\frac{K_1}{2}(x-b_1)^2 + \frac{K_2}{2}(x-b_2)^2}_{\text{linear combination of basis}} + \underbrace{\left(-K_1 b_1^2 - K_2 b_2^2 + \frac{(K_1 b_1 + K_2 b_2)^2}{K_1 + K_2}\right)}_{x\text{-independent constant}} \quad (19)$$

where b_1 and b_2 are two positive constants chosen to be smaller and larger than any possible valid bond or angle—they must satisfying $b_1 < x < b_2$ for $\forall x$ that might be observed. Here, Stage III of the espaloma pipeline would predict K_1, K_2, which would be used to compute the MM parameters (K, b) via

$$K = K_1 + K_2 \quad (20)$$

$$b = \frac{K_1 b_1 + K_2 b_2}{K_1 + K_2} \quad (21)$$

When training to fit energies, our loss function subtracts the mean from the training set energies and predicted energies for each molecule to remove the arbitrary offset constant introduced by this transformation of variables.

D.1.2 Training by potential energies

Given a training set of molecules $\{\mathcal{G}_m\}$, $m = 1, \ldots, M$, with corresponding $n = 1, \ldots, N_m$ conformational snapshots $\mathbf{x}_{m,n} \in \mathbf{R}^{(|\mathcal{G}_m| \times 3)}$ for each molecule m, and reference potential energies $\{U_{\text{ref}}(x_{m,n};;\mathcal{G}_m)\}$, the model parameters Φ_{NN} can be optimized to minimize a measure of deviation between the reference energies and model energies given by the composed force field $\Phi_{\text{FF},\Theta}$. For example, a squared loss function can be used to quadratically penalize deviations:

$$\mathcal{L}(\Phi_{\text{NN}}) = \sum_{m=1}^{M}\sum_{n=1}^{N_m} w_{m,n} \left[U_{\text{ref}}(x_{m,n};\mathcal{G}_m) - U_{\Phi_{\text{FF},\Theta}}(\mathbf{x}_{m,n};\mathcal{G}_m)\right]^2 \quad (22)$$

In this work, we took the weights $w_{m,n} = 1$, but more sophisticated weighting schemes can be used to emphasize low-energy snapshots where MM potentials are intended to be more accurate when fitting to quantum chemical datasets, as in ForceBalance [45, 46]. In a Bayesian context, the loss function in Eq. 22 would correspond to the negative log likelihood for a normal error model. Other loss or likelihood function forms may also be useful in ensuring a minority of molecules or geometries for which the MM functional form poorly reproduces quantum chemical energetics do not dominate the fit.

Additional terms can be added to the loss function to regularize the choice of parameters, such as penalizing unphysical choices (such as unphysical magnitudes or parameter regions) or to provide more useful models (such as penalizing bond vibrations with effective periods that are so short they would require extremely small timesteps).

When fitting to quantum chemical energies, an additive offset for each molecule m corresponding to the heat of formation generally cannot be accounted for using standard MM functional forms. To address this, we subtract the per-molecule mean potential energy for both predicted MM and reference quantum chemical energies in formulating the loss function, with the goal of ensuring that relative conformational energetics are accurately approximated even if the heat of formation cannot be computed.

D.1.3 Other differentiable objectives

In quantum chemical calculations, nuclear potential energy gradients are often available at very low cost once the wavefunction has been solved to compute the energy. To exploit the rich information available in these gradients [47–50] $\nabla_{\mathbf{x}} U$, it is possible to incorporate both energies and gradients into the loss function, with automatic differentiation used to compute partial derivatives of the espaloma energy function in terms of both coördinates and parameters. Additional terms penalizing deviations in more complex quantum chemical properties, such as polarizabilities or vibrational frequencies, could also be incorporated [45, 46]. In this work, however, we found that incorporation of gradient information does not significantly enhance the performance of the model. We leave this topic to future study.

In addition to incorporating configuration-dependent properties, if the goal is to build a complete molecular mechanics force field to model entire physical systems in the condensed phase, additional terms could be added to the objective function to quantify the deviation in physical properties, such as densities, dielectric constants, and enthalpies of vaporization [45, 46, 51–54], or even experimental transfer free energies.

D.1.4 Assigning espaloma parameters to molecules

In contrast with many quantum machine learning (QML) force fields that use a neural model to compute the energy and gradient for every configuration [55–57], **espaloma** uses a neural model to assign molecular mechanics parameters *once* at the beginning of a simulation, using only the chemical information about all components in a system. Once generated, the MM parameters Φ_{FF} for the system can be seamlessly ported to molecular mechanics packages that can exploit accelerated hardware [5, 58–60] to achieve high accuracy for individual systems at the same speed as traditional force fields.

E Graph neural network (GNN) architectures considered in this paper

We considered the following graph neural network architectures available in DGL [2] in this paper:

Model	Edge update ϕ^e	Edge aggregate $\rho^{e \to v}$	Node update ϕ^v
GCN	Identity	Mean	NN
EdgeConv	$\text{ReLU}(W_0(\mathbf{v}_i - \mathbf{v}_j) + W_1 \mathbf{v}_i)$	Max	Identity
GraphSAGE	Identity	Mean*	$\text{Normalize}(\text{NN}([\mathbf{v} : \mathbf{e}]))$
GIN	Identity	Sum*	$\text{NN}((1+\epsilon)\mathbf{v} + \mathbf{e})$

Table S2. Summary of representative graph neural network architectures by edge update, edge aggre-gate, and node update types. Models analyzed here include: GCN [14], EdgeConv [61], GraphSAGE [15], and GIN [13]. Other architectures evaluated—TAGCN [62] and SGC [63]—involve multi-step propagation, which could be expressed as a combination of these updates and aggregates.
*: Multiple aggregation functions studied in the referenced publication.

F Code snippets for using espaloma

F.1 Designing and training espaloma model

```
import torch, dgl, espaloma as esp

# retrieve OpenFF Gen2 Optimization Dataset
dataset = esp.data.dataset.GraphDataset.load("gen2").view(batch_size=128)

# define espaloma stage I: graph -> atom latent representation
representation = esp.nn.Sequential(
    layer=esp.nn.layers.dgl_legacy.gn("SAGEConv"), # use SAGEConv implementation in DGL
    config=[128, "relu", 128, "relu", 128, "relu"], # 3 layers, 128 units, ReLU activation
)

# define espaloma stage II and III:
# atom latent representation -> bond, angle, and torsion representation and parameters
```

```
readout = esp.nn.readout.janossy.JanossyPooling(
    in_features=128, config=[128, "relu", 128, "relu", 128, "relu"],
    out_features={              # define modular MM parameters espaloma will assign
        1: {"e": 1, "s": 1}, # atom hardness and electronegativity
        2: {"log_coefficients": 2}, # bond linear combination, enforce positive
        3: {"log_coefficients": 3}, # angle linear combination, enforce positive
        4: {"k": 6}, # torsion barrier heights (can be positive or negative)
    },
)

# compose all three espaloma stages into an end-to-end model
espaloma_model = torch.nn.Sequential(
                 representation, readout, esp.nn.readout.janossy.ExpCoefficients(),
                 esp.mm.geometry.GeometryInGraph(), esp.mm.energy.EnergyInGraph(),
                 esp.nn.readout.charge_equilibrium.ChargeEquilibrium(),
)

# define training metric
metrics = [
    esp.metrics.GraphMetric(
            base_metric=torch.nn.MSELoss(), # use mean-squared error loss
            between=['u', "u_ref"],         # between predicted and QM energies
            level="g", # compare on graph level
    )
    esp.metrics.GraphMetric(
            base_metric=torch.nn.MSELoss(), # use mean-squared error loss
            between=['q', "q_hat"],         # between predicted and reference charges
            level="n1", # compare on node level
    )
]

# fit espaloma model to training data
results = esp.Train(
    ds_tr=dataset, net=espaloma_model, metrics=metrics,
    device=torch.device('cuda:0'), n_epochs=5000,
    optimizer=lambda net: torch.optim.Adam(net.parameters(), 1e-3), # use Adam optimizer
).run()

torch.save(espaloma_model, "espaloma_model.pt") # save model
```

Listing 1. Defining and training a modular espaloma model.

F.2 Deploying espaloma model

```
# define or load a molecule of interest via the Open Force Field toolkit
from openff.toolkit.topology import Molecule
molecule = Molecule.from_smiles("CN1C=NC2=C1C(=O)N(C(=O)N2C)C")

# create an espaloma Graph object to represent the molecule of interest
import espaloma as esp
molecule_graph = esp.Graph(molecule)

# apply a trained espaloma model to assign parameters
espaloma_model = torch.load("espaloma_model.pt")
espaloma_model(molecule_graph.heterograph)

# create an OpenMM System for the specified molecule
openmm_system = esp.graphs.deploy.openmm_system_from_graph(molecule_graph)
```

Listing 2. Using a trained espaloma model to assign parameters to a small molecule.

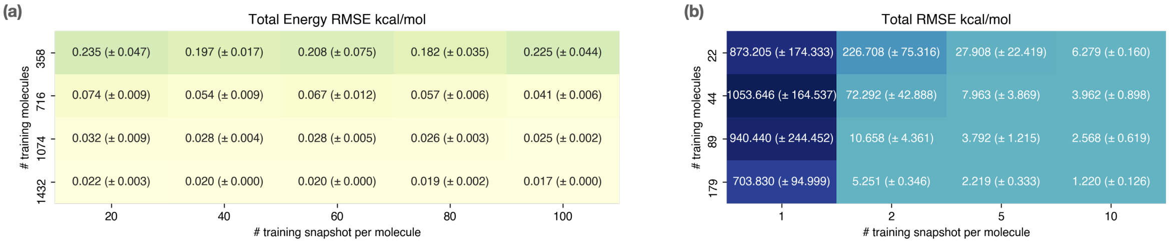

Figure S2. (a) The data efficiency of espaloma was assessed in a typical use-case regime by exploring the test set energy RMSE as a function of the number of training molecules and snapshots per molecule. The standard deviation over three fitting experiments with different random seeds is shown in parenthesis. Once a sufficiently large number of molecules are available, doubling the number of snapshots per molecule does not reduce the error as rapidly as doubling the number of molecules. (b) The data efficiency of espaloma in a data-poor regime was assessed in the same manner as Figure **??**, but for a small number of molecules and training snapshots per molecule. In the data-poor regime, increasing both the number of molecules and snapshots per molecule can deliver large decreases in test set error.

G Espaloma requires few conformations per molecule to achieve high accuracy

We examined the data efficiency of espaloma by repeating the MM fitting experiment with varying numbers of molecules and snapshots per molecule in an attempt to address whether *molecular diversity* or *conformational diversity* is more important. In the typical data regime (***Figure S2***), once sufficient conformational diversity is reached (~20 snapshots/molecule), increasing molecular diversity more effectively reduces error, though this meets with diminishing returns past a certain point. In the low data regime (***Figure S2***), both molecular and conformational diversity are important for reducing error to useful regimes, with a minimal threshold for each required to achieve reasonable errors.

H Close examination of molecular mechanics (MM) fitting experiments

Ablation study: the role of energy centering, the inclusion of torsion energies, and the inclusion of small rings in datasets in the performance of MM fitting.

We closely examined several choices which appear to have significant impact on the ability to produce high-quality generalizable models for the task of learning an MM potential: Concretely, we conduct an ablation study (Table S3) where we repeat the experiment shown in Figure 2 considering several variations of these choices:

Small rings (R): We consider whether molecules with small (3- or 4-membered) rings are included in the dataset, since these molecules assume geometries have near-degeneracies in equilibrium angles (Figure S4). **Energy centering (C):** We consider the role of energy centering in the loss function, where the average po-tential energy over all conformations for each molecule is subtracted for both reference and predicted energies in the loss function, such that only relative conformational energy errors are penalized. We evaluate the error both with and without centering. **Torsion energies (T):** We also consider whether torsion energies are included as well, since recovery of torsion profiles can be challenging due to both the near-degeneracy of parameters and phases. Notably, while MM force fields generally specify a minimal set of a few torsion periodicities with phases of 0 or π explicitly specified, we use a formulation where all periodicities $n = 1, 2, \ldots, 6$ are always fit and the sign of the torsion K parameter indicates whether the phase is 0 ($K > 0$) or π ($K < 0$).

Experiments for all combinations of these choices are shown in Table S3.

We summarize our findings as follow: First of all, best performance is only achieved when we center the predicted and reference energies in the loss function used in training, effectively training to fit only relative energies, suggesting this makes robust optimization easier. The rationalization could be found in Section D.1.1. We also examined the training trajectory of espaloma on this MM fitting task with and without the centering of predicted and reference energies (Figure S3) and noticed that centering alleviates, but not completely remedies the training difficulties of espaloma models.

Secondly, the inclusion of torsion energies deteriorates performance, especially if measured by uncen-

**R C T**	Part	$U_{\texttt{total}}$	$U_{\texttt{bond}}$	$U_{\texttt{angle}}$	$U_{\texttt{torsion}}$	$U_{\texttt{total}}$	$U_{\texttt{bond}}$	$U_{\texttt{angle}}$	$U_{\texttt{torsion}}$	k_r	b_r	k_θ	b_θ
		RMSE (kcal/mol)				Centered RMSE (kcal/mol)				MAPE			
000	test	$0.8723^{0.9334}_{0.8139}$	$1.2725^{1.4465}_{1.0575}$	$1.2291^{1.4102}_{0.9563}$		$0.7829^{0.8428}_{0.7025}$	$0.6501^{0.7308}_{0.5919}$	$0.4553^{0.4860}_{0.4245}$		$0.1238^{0.1265}_{0.1206}$	$0.0026^{0.0027}_{0.0025}$	$0.0733^{0.0744}_{0.0722}$	$0.0067^{0.0068}_{0.0066}$
	train	$0.9183^{0.9889}_{0.8343}$	$1.3159^{1.5514}_{1.0655}$	$1.2844^{1.5360}_{1.0052}$		$0.8681^{0.9330}_{0.7993}$	$0.7209^{0.7771}_{0.6639}$	$0.4815^{0.5097}_{0.4431}$		$0.1293^{0.1320}_{0.1253}$	$0.0027^{0.0028}_{0.0027}$	$0.0726^{0.0736}_{0.0712}$	$0.0071^{0.0073}_{0.0069}$
001	test	$4.4033^{4.6173}_{4.1317}$	$13.7162^{14.5515}_{12.9510}$	$7.9210^{8.4048}_{7.3953}$	$0.0363^{0.0384}_{0.0345}$	$3.5238^{3.6590}_{3.3984}$	$3.0956^{3.2187}_{2.9908}$	$1.7086^{1.7619}_{1.6367}$	$0.0015^{0.0017}_{0.0014}$	$1.0000^{1.0000}_{1.0000}$	$0.0720^{0.0740}_{0.0701}$	$0.4734^{0.4763}_{0.4712}$	$0.0136^{0.0138}_{0.0134}$
	train	$4.2372^{4.5129}_{3.9854}$	$13.2695^{14.2219}_{12.1669}$	$8.0467^{8.6800}_{7.4667}$	$0.0353^{0.0379}_{0.0330}$	$3.5041^{3.6277}_{3.3614}$	$3.0241^{3.1645}_{2.9115}$	$1.7425^{1.8182}_{1.6659}$	$0.0016^{0.0017}_{0.0014}$	$1.0000^{1.0000}_{1.0000}$	$0.0705^{0.0720}_{0.0686}$	$0.4803^{0.4831}_{0.4778}$	$0.0137^{0.0139}_{0.0135}$
010*	test	$0.0490^{0.0585}_{0.0369}$	$0.0206^{0.0262}_{0.0158}$	$0.0429^{0.0497}_{0.0362}$		$0.0175^{0.0187}_{0.0163}$	$0.0130^{0.0140}_{0.0121}$	$0.0117^{0.0127}_{0.0105}$		$0.0016^{0.0017}_{0.0016}$	$0.0001^{0.0001}_{0.0001}$	$0.0020^{0.0020}_{0.0019}$	$0.0006^{0.0007}_{0.0006}$
	train	$0.0394^{0.0478}_{0.0315}$	$0.0174^{0.0206}_{0.0142}$	$0.0332^{0.0394}_{0.0271}$		$0.0169^{0.0178}_{0.0160}$	$0.0128^{0.0135}_{0.0118}$	$0.0113^{0.0121}_{0.0103}$		$0.0016^{0.0016}_{0.0016}$	$0.0001^{0.0001}_{0.0001}$	$0.0020^{0.0021}_{0.0020}$	$0.0006^{0.0006}_{0.0005}$
011	test	$11.0688^{12.8267}_{8.8832}$	$0.0240^{0.0284}_{0.0204}$	$0.1801^{0.2122}_{0.1535}$	$0.0174^{0.0203}_{0.0145}$	$0.0346^{0.0369}_{0.0324}$	$0.0168^{0.0180}_{0.0157}$	$0.0270^{0.0307}_{0.0226}$	$0.0001^{0.0001}_{0.0001}$	$0.0020^{0.0021}_{0.0020}$	$0.0001^{0.0001}_{0.0001}$	$0.0020^{0.0021}_{0.0020}$	$0.0009^{0.0009}_{0.0008}$
	train	$11.7819^{14.0593}_{9.4086}$	$0.0190^{0.0203}_{0.0179}$	$0.1899^{0.2335}_{0.1545}$	$0.0185^{0.0216}_{0.0152}$	$0.0335^{0.0357}_{0.0302}$	$0.0161^{0.0173}_{0.0150}$	$0.0284^{0.0321}_{0.0238}$	$0.0001^{0.0001}_{0.0001}$	$0.0020^{0.0021}_{0.0020}$	$0.0001^{0.0001}_{0.0001}$	$0.0020^{0.0021}_{0.0020}$	$0.0009^{0.0009}_{0.0008}$
100	test	$0.6237^{0.7049}_{0.5459}$	$0.5224^{0.5707}_{0.4774}$	$0.6660^{0.7498}_{0.5843}$		$0.4970^{0.5526}_{0.4483}$	$0.3380^{0.3614}_{0.3176}$	$0.4654^{0.5205}_{0.4084}$		$0.0469^{0.0479}_{0.0459}$	$0.0012^{0.0013}_{0.0012}$	$0.0645^{0.0662}_{0.0624}$	$0.0111^{0.0117}_{0.0107}$
	train	$0.6615^{0.7584}_{0.5706}$	$0.6199^{0.6905}_{0.5480}$	$0.6939^{0.7702}_{0.6107}$		$0.5693^{0.6494}_{0.4963}$	$0.3916^{0.4304}_{0.3473}$	$0.5293^{0.6160}_{0.4489}$		$0.0482^{0.0493}_{0.0473}$	$0.0013^{0.0013}_{0.0013}$	$0.0659^{0.0677}_{0.0637}$	$0.0112^{0.0117}_{0.0108}$
101	test	$4.7719^{4.9088}_{4.6354}$	$9.5963^{9.9684}_{9.1500}$	$23.1972^{24.2192}_{22.2922}$	$0.0501^{0.0519}_{0.0480}$	$4.3643^{4.4498}_{4.2759}$	$2.6994^{2.7756}_{2.6075}$	$3.6263^{3.6948}_{3.5347}$	$0.0020^{0.0022}_{0.0019}$	$0.8410^{0.8440}_{0.8386}$	$0.0263^{0.0267}_{0.0259}$	$1.0000^{1.0000}_{1.0000}$	$0.9287^{0.9294}_{0.9282}$
	train	$4.8043^{4.9617}_{4.6731}$	$9.3807^{9.8568}_{8.8160}$	$23.2491^{24.1563}_{22.4420}$	$0.0495^{0.0514}_{0.0481}$	$4.4050^{4.5169}_{4.2726}$	$2.6971^{2.7786}_{2.6393}$	$3.5734^{3.6850}_{3.4918}$	$0.0019^{0.0021}_{0.0018}$	$0.8404^{0.8428}_{0.8371}$	$0.0267^{0.0272}_{0.0263}$	$1.0000^{1.0000}_{1.0000}$	$0.9281^{0.9288}_{0.9275}$
110	test	$89.0006^{102.2105}_{70.4894}$	$0.0384^{0.0440}_{0.0339}$	$88.9808^{107.0304}_{64.5673}$		$0.0223^{0.0257}_{0.0200}$	$0.0246^{0.0269}_{0.0221}$	$0.0278^{0.0310}_{0.0247}$		$0.0018^{0.0019}_{0.0018}$	$0.0001^{0.0001}_{0.0001}$	$0.0033^{0.0035}_{0.0032}$	$0.0074^{0.0089}_{0.0064}$
	train	$79.6927^{99.1904}_{62.4512}$	$0.1155^{0.1924}_{0.0351}$	$79.6589^{98.9583}_{59.2236}$		$0.0209^{0.0236}_{0.0185}$	$0.0815^{0.1566}_{0.0243}$	$0.0814^{0.1351}_{0.0264}$		$0.0018^{0.0020}_{0.0017}$	$0.0001^{0.0001}_{0.0001}$	$0.0031^{0.0033}_{0.0029}$	$0.0062^{0.0076}_{0.0051}$
111	test	$99.6155^{118.2089}_{75.0240}$	$0.0586^{0.0661}_{0.0520}$	$91.7758^{115.4096}_{71.2381}$	$0.0245^{0.0270}_{0.0222}$	$0.0297^{0.0352}_{0.0262}$	$0.0327^{0.0371}_{0.0282}$	$0.0438^{0.0492}_{0.0398}$	$0.0000^{0.0001}_{0.0000}$	$0.0017^{0.0018}_{0.0016}$	$0.0001^{0.0001}_{0.0001}$	$0.0034^{0.0036}_{0.0032}$	$0.0077^{0.0087}_{0.0066}$
	train	$90.1890^{113.6430}_{70.8691}$	$0.1682^{0.2789}_{0.0543}$	$81.6664^{98.1073}_{64.1198}$	$0.0264^{0.0290}_{0.0236}$	$0.0279^{0.0299}_{0.0258}$	$0.1015^{0.1693}_{0.0311}$	$0.1043^{0.1679}_{0.0440}$	$0.0001^{0.0001}_{0.0000}$	$0.0017^{0.0018}_{0.0017}$	$0.0001^{0.0001}_{0.0001}$	$0.0033^{0.0035}_{0.0031}$	$0.0065^{0.0075}_{0.0055}$

Table S3. Best performance of MM fitting on PhAlkEthOH dataset only when centering is deployed and torsion energy and small rings are excluded. A comparison of espaloma MM fitting experiments on PhAlkEthOH dataset with/without fitting torsions, centering energies, and including three- and four-membered rings. The **R C T** col-umn summarizes the experimental conditions: **R** indicates whether small (3- and 4-membered) rings were included in the dataset (1 small rings included, 0 excluded); (**C**) indicates whether the potential energies for sampled snapshots of each molecule were *centered* by subtracting off the mean energy for that molecule in the loss function (1 centered, 0 uncentered); (**T**) indicates whether torsion energies were included in the fitting (1 included, 0 excluded). The rest of the experimental setting is identical with Figure 2. Force field parameters (Φ_{FF}): k_r: bond force constant; b_r: equilibrium bond length; k_θ: angle force constant; b_θ: equilibrium angle value. The root mean squared error (RMSE) and correlation coefficient (R^2) between reference and predicted MM energies, as well as mean absolute percentage error (MAPE) (as a fraction rather as a percentage, i.e. not multiplied by 100), and correlation coefficient (R^2) between reference and predicted force field parameters. The sub- and superscripts report the 95% confidence interval of each statistics estimated from 1000 bootstrapped replicates on the molecule set. *: Same experiment as Figure 2.

tered RMSE. This could be attributed to the fact that we use a slightly different functional form to express torsion energies than reference legacy force fields.

Lastly, with energy centering, including three- and four-membered rings in our training set results in very large uncentered RMSE without drastically changing the centered RMSE (relative conformational energy errors). We study this effect in detail in the next section.

Case Study: Parametrization of Cyclopropane

We use the model trained on PhAlkEthOH dataset with the inclusion of small rings but without torsion energies (experiment 110 in Table S3) and apply this model to cyclopropane (SMILES: `C1CC1`) As shown in (b) in Figure S4, when trained with centered energy loss function, espaloma assigned equilibrium angle value $\theta_0 \approx 122.1°$ to the CCC angle in cyclopropane while the equilibrium angle is $60°$ in the reference GAFF-1.81 force field. With a shift in angle force constant, while this would drastically change the energies of individual CCC angles, the sum of energies of the three CCC angles (whose angle values would sum up to $180°$) would remain very close to the reference value up to a constant. We furthermore compared (in Figure S4-(e)) the sum of CCC angle energies in these two parametrizations on a wide range of geometries of cyclopropane with three CCC angles θ_0, θ_1, θ_2 satisfying $50° < \Theta_0 < 70°$, $-10° < \theta_1 - \theta_2 < 10°$ and noticed that the difference is always a constant with fluctuations within 1 kcal/mol.

I Espaloma can easily parameterize complex heterogeneous biomolecular systems

Because biomolecular systems of interest are often highly heterogeneous environments, it is often practically incredibly difficult to model systems that consist of more than the simplest protein-ligand-solvent combinations. For example, the latest Amber 20 release [64] recommends a set of independently developed (but loosely coupled) set of force fields and complex parameterization machinery for proteins [21], DNA [65],

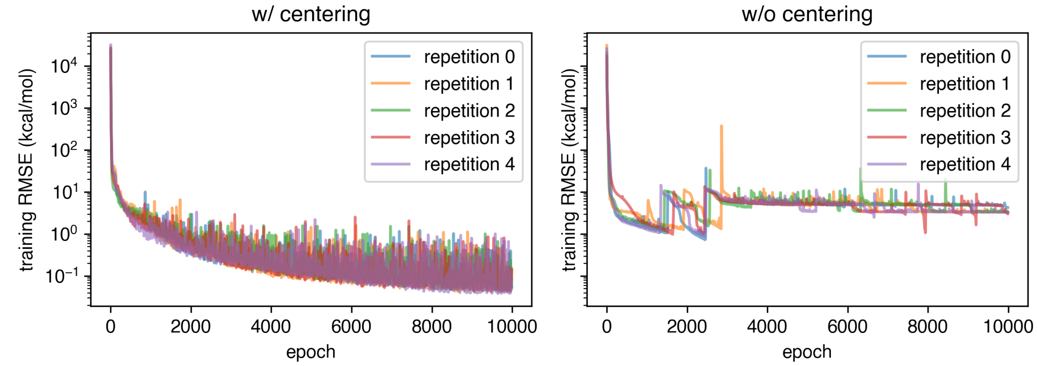

w/ centering					w/o centering				
Rep.	ES Epoch	RMSE at ES (kcal/mol)			Rep.	ES Epoch	RMSE at ES (kcal/mol)		
		Train	Validation	Test			Train	Validation	Test
0	9602	0.0456	0.0523	0.0834	0	2447	0.7331	0.6937	0.7705
1	8938	0.0381	0.0500	0.0815	1	1902	1.2449	1.1950	1.1910
2	9967	0.0438	0.0503	0.0545	2	1424	1.1646	1.1351	1.1537
3	9696	0.0411	0.0342	0.0714	3	2422	0.9849	0.9121	0.9805
4	9879	0.0377	0.0470	0.0714	4	2446	0.8727	0.8006	0.8460

Figure S3. espaloma models are difficult to train, especially when attempting to fit to absolute (rather than relative) conformation energies. We compare the training trajectory of espaloma without torsion energy on PhAlkEthOH dataset excluding three- and four-membered rings with and without centering (experiments 010 and 000 in Table S3). Example training trajectories with the same setting as Figure 2. Various repetitions are plotted in different colors.

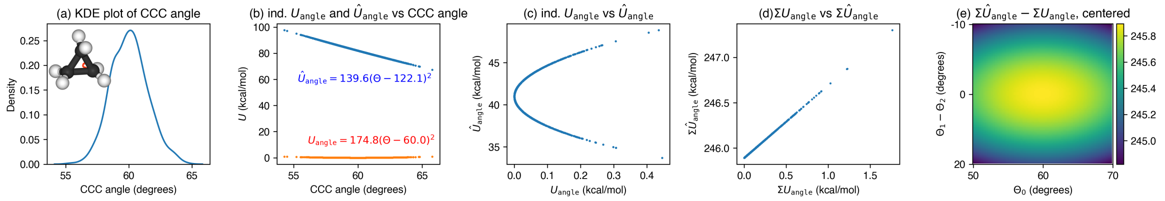

Figure S4. A comparison of reference and espaloma-learned parametrization of cyclopropane. (a): KDE plot of CCC angle in cyclopropane. (b): **Individual** reference and predicted angle energy ($U_{\mathtt{angle}}$ and $\hat{U}_{\mathtt{angle}}$, energy of each CCC angle) plotted against CCC angle values in the snapshots generated. (c): Predicted plotted against reference **individual** CCC energy. (d): Predicted plotted against reference **sum** CCC energy. (e): Distribution of difference between reference $\Sigma U_{\mathtt{angle}}$ and predicted $\Sigma \hat{U}_{\mathtt{angle}}$ **sum** CCC energy.

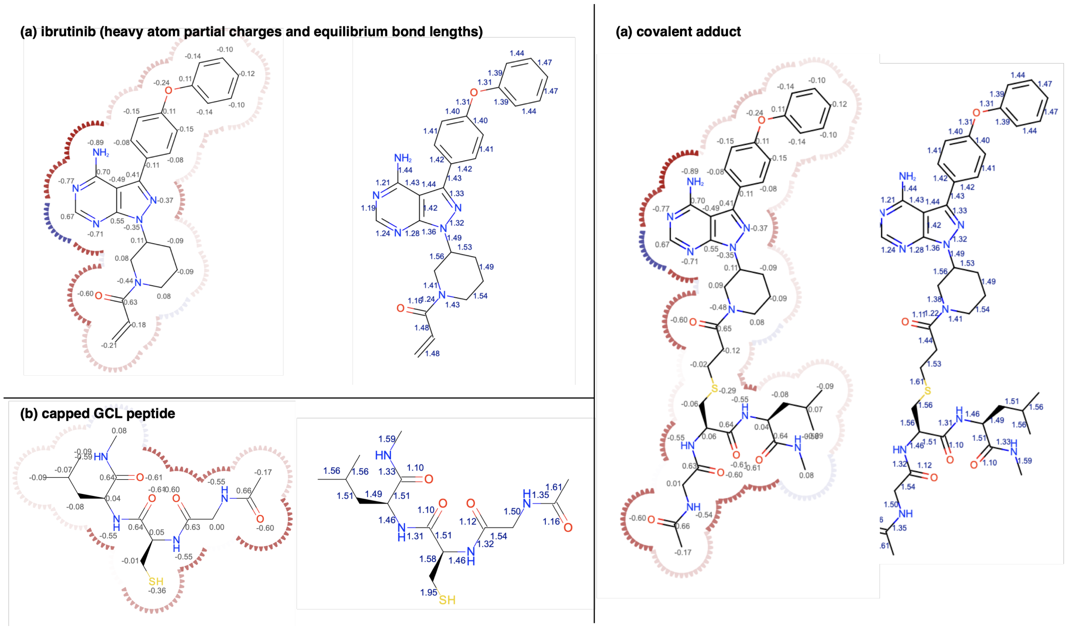

Figure S5. espaloma parametrizes covalent inhibitor adduct system. Heavy atom bond equilibrium length (in angstrom) and partial charge (in elemental charge) prediction for (a) ibrutinib; (b) capped GCL peptide; and (c) their covalent adduct.

RNA [66], water [23], monovalent [24, 67] and divalent [68–70] counterions, lipids [71], carbohydrates [72], glycoconjugates [73, 74], small molecules [9, 10], post-translational modifications [75], and nucleic acid modifications [76], collectively representing hundreds of human-years of effort. Despite this, even seemingly simple common tasks—such as modeling irreversible covalent inhibitors—represent a mind-bending technical challenge for parameterization [77].

Espaloma operates on only the chemical graph of components of the system, which enables it to solve many issues with these legacy approaches: In generating parameters, there is no practical difference between any of the biopolymer, biomolecule, organic molecule, solvent, ion, or covalently modified species that previously required enormous effort to separately build parameter sets to cover; espaloma will simply interpolate the parameters from observed examples in a continuous manner. Instead of separately curating distinct datasets and philosophies for parameterizing each of these different classes of chemical species, extending an espaloma model to a new class of chemical species is simply a matter of extending the training datasets of quantum chemical and/or physical property data. As we have seen, building an elementary model capable of simulating solvated protein-ligand complexes simply required incorporating quantum chemical datasets used to parameterize a small molecule force field alongside quantum chemical data for short peptides.

This espaloma model should also provide sufficient coverage to model more complex protein-ligand covalent conjugates, since the relevant chemistry to model this complex already exists in the training dataset. To demonstrate that this leads to stable, sensible parameters, we considered the pre-reactive form of the covalent kinase inhibitor ibrutinib and the terminally-blocked form of the Val-Cys-Gly sequence that it reacts with in its target kinase BTK (Figure S5, left). Espaloma is able to rapidly parameterize the covalent conjugate of ibrutinib to this BTK target sequence, resulting in minor changes to parameters and partial charges around the covalent warhead, but minimally perturbing the remainder of the system (Figure S5, right). Unlike legacy approaches to parameterizing covalently-modified residues, no expensive parameter or charge refinement procedure was needed for this task.

J Espaloma approximates quantum chemical minima

We examined whether the espaloma force field described in Section 3 not only quantitatively reproduced quantum chemical equilibrium conformational energetics, but was able to also qualitatively preserve quan-

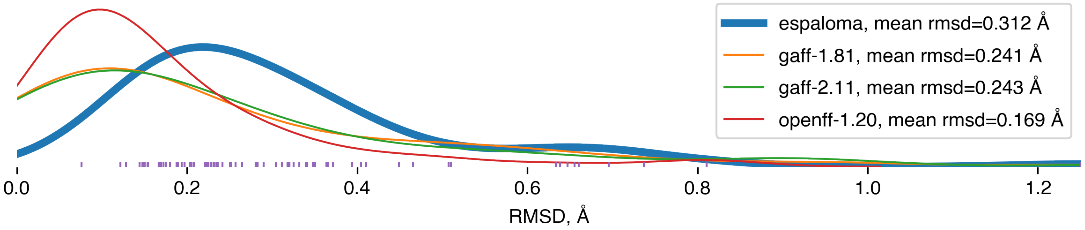

Figure S6. Espaloma-derived molecular mechanics force fields preserve the location of quantum chem-ical minima. Kernel density estimate (KDE) plot of root mean square deviation (RMSD) between quantum chemical min-ima and MM-optimized minima using parameters from either espaloma-generated molecular mechanics or legacy force fields.

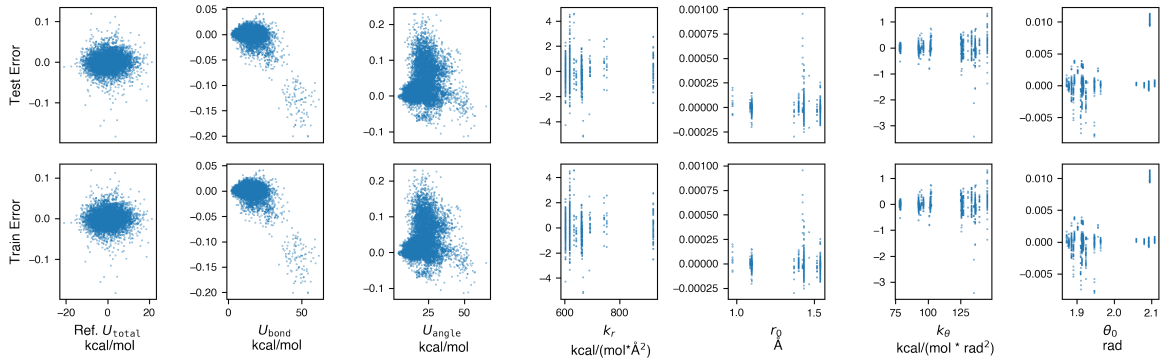

Figure S7. Espaloma models fitted to a legacy molecular mechanics potential show small relative er-rors. Signed energy and parameter errors for the experiment shown in Figure 3, where an espaloma model was trained to reproduce GAFF 1.81 energies, are shown. Relative error magnitudes are small, and the signed errors show little relative systematic bias in recovered parameters.

tum chemical local minima. To assess this, we initiated MM energy minimizations from QM-optimized con-formations from the test set of OpenFF Gen 2 Optimization dataset. Specifically, we selected the minimum quantum chemical energy snapshot for each molecule in the Parsley dataset and minimized with espaloma-derived force field, OpenFF 1.2.0, GAFF 1.81, or GAFF 2.11 MM force fields using an L-BFGS optimizer with a 0.1 kJ/mol convergence tolerance. Figure S6 shows a kernel density estimate (KDE) of the root mean squared distance (RMSD) from the quantum chemical minimum produced by each force field. We notice that despite the competitive results in the energy prediction tasks, there is space for improvements when it comes to recover the QM minima. We expect that incorporating forces in the training process could greatly alleviate this pathology. We leave this for future study.

K Additional figures

L Proof that Janossy pooling is sufficiently expressive

Now we prove that such formulation is expressive enough to distinguish bonds consisting of distinct atoms. An equivalent proof for angles and torsions follows similarly.

Lemma 1. *There exists a neural function* NN_r*, such that,*

$$\mathrm{NN}_r([h_{v_i} : h_{v_j}]) + \mathrm{NN}_r([h_{v_j} : h_{v_i}]) = \mathrm{NN}_r([h_{v_k} : h_{v_l}]) + \mathrm{NN}_r([h_{v_k} : h_{v_l}])$$

if and only if $h_{v_i} = h_{v_l}$ *and* $h_{v_j} = h_{v_k}$ *or* $h_{v_i} = h_{v_l}$ *and* $h_{v_j} = h_{v_k}$*.*

Proof. We first prove that there is a *function* f_r satisfying the condition in Lemma 1. With finite possible initial conditions and finite rounds of message passing, the possible values of h_v is finite (corresponding to

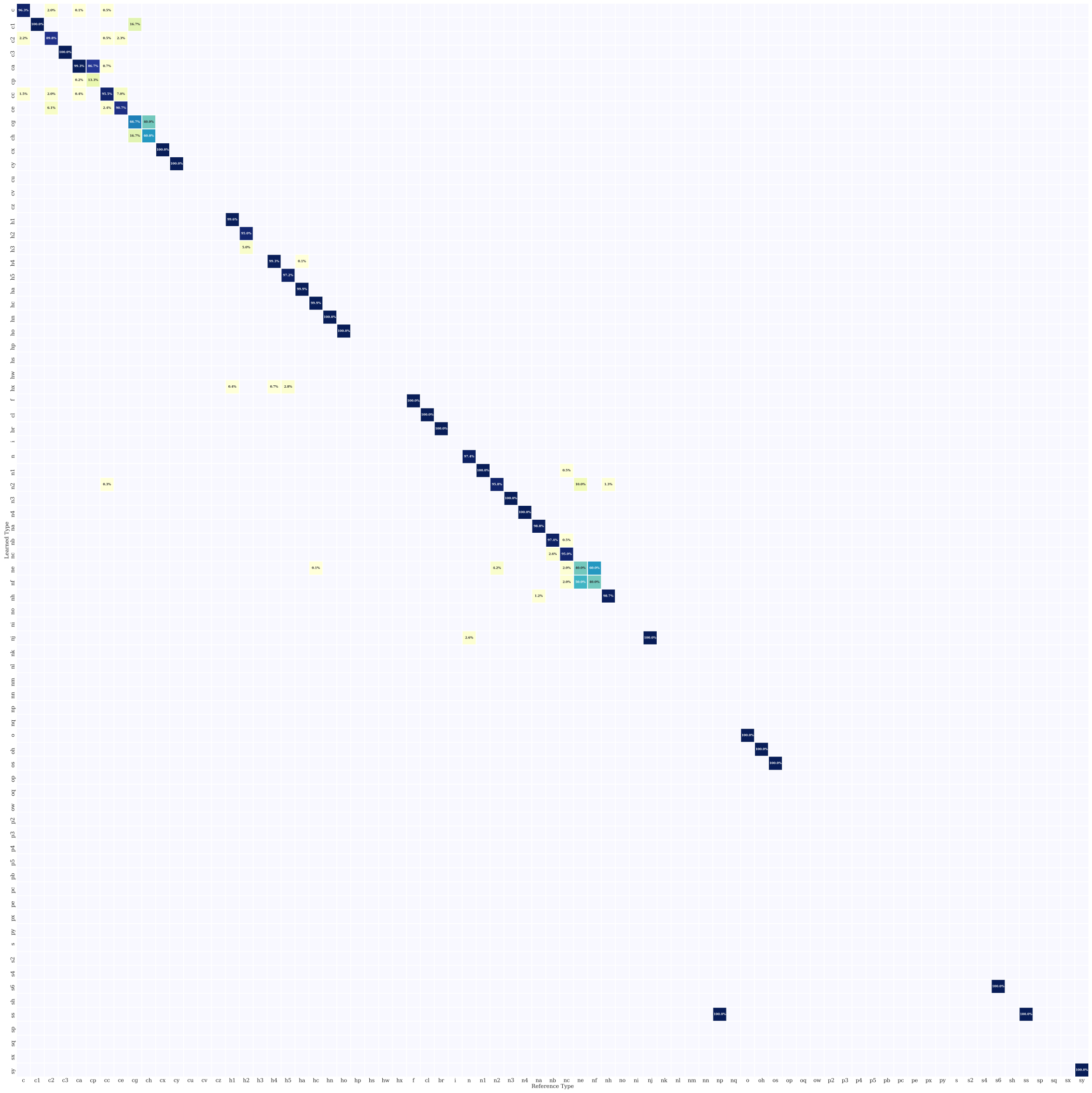

Figure S8. Confusion Matrix: Reference vs Learned Atom Types. Continuation of the confusion matrix shown in Figure 2, to include not just carbon types. The blank entries are because the dataset does not cover some of the rare atom types.

the finite number of unique labels in Weisfeiler-Leman [78] test). We use c to denote the maximum value of the hash of h_v:

$$c = \max\{\text{hash}(h_v)\} \tag{23}$$

Thus, one example of such $f_r : \mathbb{R}^D \to \mathbb{N}$ satisfying the condition in Lemma 1 is:

$$f_r(h_{v_i}, h_{v_j}) = \begin{cases} 0, \text{hash}(h_{v_i}) \geq \text{hash}(h_{v_j}); \\ (c+1)\,\text{hash}(h_{v_i}) + \text{hash}(h_{v_i}), \text{hash}(h_{v_i}) < \text{hash}(h_{v_j}). \end{cases} \tag{24}$$

Following the universal approximation theorem [79, 80], there exists a neural function NN_r that approximates f_r arbitrarily well and thus satisfies the condition in Lemma 1. □

References

[1] Adam Paszke, Sam Gross, Soumith Chintala, Gregory Chanan, Edward Yang, Zachary DeVito, Zeming Lin, Alban Desmaison, Luca Antiga, and Adam Lerer. Automatic differentiation in pytorch. 2017.

[2] Minjie Wang, Da Zheng, Zihao Ye, Quan Gan, Mufei Li, Xiang Song, Jinjing Zhou, Chao Ma, Lingfan Yu, Yu Gai, et al. Deep graph library: A graph-centric, highly-performant package for graph neural networks. *arXiv preprint arXiv:1909.01315*, 2019.

[3] David L Mobley, Caitlin C Bannan, Andrea Rizzi, Christopher I Bayly, John D Chodera, Victoria T Lim, Nathan M Lim, Kyle A Beauchamp, Michael R Shirts, Michael K Gilson, et al. Open force field consortium: Escaping atom types using direct chemical perception with smirnoff v0. 1. *BioRxiv*, page 286542, 2018.

[4] Jeff Wagner, David L. Mobley, Caitlin Bannan, John Chodera, Andrea Rizzi, Matt Thompson, Josh Horton, David Dotson, Jaime Rodríguez-Guerra, Camila, Christopher Bayly, JoshHorton, trevorgokey, Nathan M. Lim, Victoria Lim, Pavan Behara, SimonBoothroyd, Sukanya Sasmal, Daniel Smith, Lee-Ping, and Yutong Zhao. openforcefield/openforcefield: 0.7.2 Bugfix and minor feature release, September 2020. URL https://doi.org/10.5281/zenodo.4057038.

[5] Peter Eastman, Jason Swails, John D Chodera, Robert T McGibbon, Yutong Zhao, Kyle A Beauchamp, Lee-Ping Wang, Andrew C Simmonett, Matthew P Harrigan, Chaya D Stern, et al. Openmm 7: Rapid development of high performance algorithms for molecular dynamics. *PLoS computational biology*, 13(7):e1005659, 2017.

[6] An informal amber small molecule force field: parm@frosst, 2010. URL http://www.ccl.net/cca/data/parm_at_Frosst/.

[7] Trevor Gokey. Openff sandbox cho phalkethoh v1.0, 2020. URL https://github.com/openforcefield/qca-dataset-submission/tree/master/submissions/2020-09-18-OpenFF-Sandbox-CHO-PhAlkEthOH.

[8] Junmei Wang, Romain M. Wolf, James W. Caldwell, Peter A. Kollman, and David A. Case. Development and testing of a general amber force field. *Journal of Computational Chemistry*, 25(9):1157–1174, 2004. doi: 10.1002/jcc.20035. URL https://onlinelibrary.wiley.com/doi/abs/10.1002/jcc.20035.

[9] Junmei Wang, Romain M Wolf, James W Caldwell, Peter A Kollman, and David A Case. Development and testing of a general amber force field. *Journal of computational chemistry*, 25(9):1157–1174, 2004.

[10] Junmei Wang, Wei Wang, Peter A Kollman, and David A Case. Automatic atom type and bond type perception in molecular mechanical calculations. *Journal of molecular graphics and modelling*, 25(2):247–260, 2006.

[11] Daniel GA Smith, Doaa Altarawy, Lori A Burns, Matthew Welborn, Levi N Naden, Logan Ward, Sam Ellis, Benjamin P Pritchard, and T Daniel Crawford. The molssi qcarchive project: An open-source platform to compute, organize, and share quantum chemistry data. *Wiley Interdisciplinary Reviews: Computational Molecular Science*, page e1491, 2020.

[12] Diederik P Kingma and Jimmy Ba. Adam: A method for stochastic optimization. *arXiv preprint arXiv:1412.6980*, 2014.

[13] Keyulu Xu, Weihua Hu, Jure Leskovec, and Stefanie Jegelka. How powerful are graph neural networks? *arXiv preprint arXiv:1810.00826*, 2018.

[14] Thomas N. Kipf and Max Welling. Semi-supervised classification with graph convolutional networks. *CoRR*, abs/1609.02907, 2016. URL http://arxiv.org/abs/1609.02907.

[15] Will Hamilton, Zhitao Ying, and Jure Leskovec. Inductive representation learning on large graphs. In *Advances in neural information processing systems*, pages 1024–1034, 2017.

[16] Felix Wu, Tianyi Zhang, Amauri H. Souza Jr., Christopher Fifty, Tao Yu, and Kilian Q. Weinberger. Simplifying graph convolutional networks. *CoRR*, abs/1902.07153, 2019. URL http://arxiv.org/abs/1902.07153.

[17] John Chodera, Andrea Rizzi, Levi Naden, Kyle Beauchamp, Patrick Grinaway, Josh Fass, Alex Wade, Bas Rustenburg, Gregory A. Ross, Andreas Krämer, Hannah Bruce Macdonald, Jaime Rodríguez-Guerra, Mike Henry, Andy Simmonett, David W.H. Swenson, Iván Pulido, hb0402, Sander Roet, Mark J. Williamson, SimonBoothroyd, Ana Silveira, and dominicrufa. choderalab/openmmtools: Bugfix release v0.21.2, February 2022. URL https://doi.org/10.5281/zenodo.6260174.

[18] John Chodera, Andrea Rizzi, Levi Naden, Kyle Beauchamp, Patrick Grinaway, Josh Fass, Alex Wade, Bas Rustenburg, Gregory A. Ross, Andreas Krämer, Hannah Bruce Macdonald, Jaime Rodríguez-Guerra, dominicrufa, Andy Simmonett, David W. H. Swenson, hb0402, Mike Henry, Sander Roet, and Ana Silveira. Choderalab/openmmtools: 0.20.3 Bugfix Release. Zenodo, March 2021.

[19] Lingle Wang, Yujie Wu, Yuqing Deng, Byungchan Kim, Levi Pierce, Goran Krilov, Dmitry Lupyan, Shaughnessy Robinson, Markus K Dahlgren, Jeremy Greenwood, et al. Accurate and reliable prediction of relative ligand binding potency in prospective drug discovery by way of a modern free-energy calculation protocol and force field. *Journal of the American Chemical Society*, 137(7):2695–2703, 2015.

[20] David F. Hahn and Jeff Wagner. openforcefield/protein-ligand-benchmark: 0.1.2. Release to create Zenodo record, May 2021. URL https://doi.org/10.5281/zenodo.4813735.

[21] James A Maier, Carmenza Martinez, Koushik Kasavajhala, Lauren Wickstrom, Kevin E Hauser, and Carlos Simmerling. ff14sb: improving the accuracy of protein side chain and backbone parameters from ff99sb. *Journal of chemical theory and computation*, 11(8):3696–3713, 2015.

[22] John Chodera, Rafal Wiewiora, Chaya Stern, and peastman. openmm/openmm-forcefields: Fix GAFF AM1-BCC charging bug for some molecules, January 2020. URL https://doi.org/10.5281/zenodo.3627391.

[23] William L Jorgensen, Jayaraman Chandrasekhar, Jeffry D Madura, Roger W Impey, and Michael L Klein. Comparison of simple potential functions for simulating liquid water. *The Journal of chemical physics*, 79(2):926–935, 1983.

[24] In Suk Joung and Thomas E Cheatham III. Determination of alkali and halide monovalent ion parameters for use in explicitly solvated biomolecular simulations. *The journal of physical chemistry B*, 112(30):9020–9041, 2008.

[25] Huafeng Xu. Optimal measurement network of pairwise differences. *Journal of Chemical Information and Modeling*, 59(11):4720–4728, 2019.

[26] Hannah Bruce Macdonald, dfhahn, Mike Henry, John Chodera, David Dotson, William Glass, and Iván Pulido. openforcefield/openff-arsenic: v0.2.1, February 2022. URL https://doi.org/10.5281/zenodo.6210305.

[27] John D Chodera and Michael R Shirts. Replica exchange and expanded ensemble simulations as gibbs sampling: Simple improvements for enhanced mixing. *The Journal of chemical physics*, 135(19):194110, 2011.

[28] Benedict Leimkuhler and Charles Matthews. Efficient molecular dynamics using geodesic integration and solvent–solute splitting. *Proceedings of the Royal Society A: Mathematical, Physical and Engineering Sciences*, 472(2189): 20160138, 2016.

[29] Jon R Maple, M-J Hwang, Thomas P. Stockfisch, Uri Dinur, Marvin Waldman, Carl S Ewig, and Arnold T. Hagler. Derivation of class ii force fields. i. methodology and quantum force field for the alkyl functional group and alkane molecules. *Journal of Computational Chemistry*, 15(2):162–182, 1994.

[30] Mj J Hwang, TP Stockfisch, and AT Hagler. Derivation of class ii force fields. 2. derivation and characterization of a class ii force field, cff93, for the alkyl functional group and alkane molecules. *Journal of the American Chemical Society*, 116(6):2515–2525, 1994.

[31] JR Maple, M-J Hwang, TP Stockfisch, and AT Hagler. Derivation of class ii force fields. iii. characterization of a quantum force field for alkanes. *Israel Journal of Chemistry*, 34(2):195–231, 1994.

[32] Zhengwei Peng, Carl S Ewig, Ming-Jing Hwang, Marvin Waldman, and Arnold T Hagler. Derivation of class ii force fields. 4. van der waals parameters of alkali metal cations and halide anions. *The Journal of Physical Chemistry A*, 101 (39):7243–7252, 1997.

[33] JR Maple, M-J Hwang, Karl James Jalkanen, Thomas P Stockfisch, and Arnold T Hagler. Derivation of class ii force fields: V. quantum force field for amides, peptides, and related compounds. *Journal of computational chemistry*, 19 (4):430–458, 1998.

[34] Pnina Dauber-Osguthorpe and Arnold T Hagler. Biomolecular force fields: where have we been, where are we now, where do we need to go and how do we get there? *Journal of computer-aided molecular design*, 33(2):133–203, 2019.

[35] Arnold T Hagler. Force field development phase ii: Relaxation of physics-based criteria… or inclusion of more rigorous physics into the representation of molecular energetics. *Journal of computer-aided molecular design*, 33(2): 205–264, 2019.

[36] Robert B Best, Xiao Zhu, Jihyun Shim, Pedro EM Lopes, Jeetain Mittal, Michael Feig, and Alexander D MacKerell Jr. Optimization of the additive charmm all-atom protein force field targeting improved sampling of the backbone ϕ, ψ and side-chain χ1 and χ2 dihedral angles. *Journal of chemical theory and computation*, 8(9):3257–3273, 2012.

[37] Jérôme Delhommelle and Philippe Millié. Inadequacy of the lorentz-berthelot combining rules for accurate predictions of equilibrium properties by molecular simulation. *Molecular Physics*, 99(8):619–625, 2001.

[38] Marvin Waldman and Arnold T Hagler. New combining rules for rare gas van der waals parameters. *Journal of computational chemistry*, 14(9):1077–1084, 1993.

[39] Christopher M Baker, Pedro EM Lopes, Xiao Zhu, Benoît Roux, and Alexander D MacKerell Jr. Accurate calculation of hydration free energies using pair-specific lennard-jones parameters in the charmm drude polarizable force field. *Journal of chemical theory and computation*, 6(4):1181–1198, 2010.

[40] David L Mobley, Caitlin C Bannan, Andrea Rizzi, Christopher I Bayly, John D Chodera, Victoria T Lim, Nathan M Lim, Kyle A Beauchamp, David R Slochower, Michael R Shirts, et al. Escaping atom types in force fields using direct chemical perception. *Journal of chemical theory and computation*, 14(11):6076–6092, 2018.

[41] Kenno Vanommeslaeghe and Alexander D MacKerell Jr. Automation of the charmm general force field (cgenff) i: bond perception and atom typing. *Journal of chemical information and modeling*, 52(12):3144–3154, 2012.

[42] Kenno Vanommeslaeghe, E Prabhu Raman, and Alexander D MacKerell Jr. Automation of the charmm general force field (cgenff) ii: assignment of bonded parameters and partial atomic charges. *Journal of chemical information and modeling*, 52(12):3155–3168, 2012.

[43] Peter W Battaglia, Jessica B Hamrick, Victor Bapst, Alvaro Sanchez-Gonzalez, Vinicius Zambaldi, Mateusz Malinowski, Andrea Tacchetti, David Raposo, Adam Santoro, Ryan Faulkner, et al. Relational inductive biases, deep learning, and graph networks. *arXiv preprint arXiv:1806.01261*, 2018.

[44] Kenno Vanommeslaeghe, Mingjun Yang, and Alexander D. MacKerell Jr. Robustness in the fitting of molecular mechanics parameters. *Journal of Computational Chemistry*, 36(14):1083–1101, 2015. doi: https://doi.org/10.1002/jcc.23897. URL https://onlinelibrary.wiley.com/doi/abs/10.1002/jcc.23897.

[45] Lee-Ping Wang, Jiahao Chen, and Troy Van Voorhis. Systematic parametrization of polarizable force fields from quantum chemistry data. *Journal of chemical theory and computation*, 9(1):452–460, 2013.

[46] Lee-Ping Wang, Todd J Martinez, and Vijay S Pande. Building force fields: An automatic, systematic, and reproducible approach. *The journal of physical chemistry letters*, 5(11):1885–1891, 2014.

[47] Anders S. Christensen and O. Anatole von Lilienfeld. On the role of gradients for machine learning of molecular energies and forces, 2020.

[48] Stefan Chmiela, Huziel E. Sauceda, Igor Poltavsky, Klaus-Robert Müller, and Alexandre Tkatchenko. sgdml: Constructing accurate and data efficient molecular force fields using machine learning. *Computer Physics Communications*, 240: 38–45, 2019. doi: 10.1016/j.cpc.2019.02.007.

[49] Huziel E. Sauceda, Stefan Chmiela, Igor Poltavsky, Klaus-Robert Müller, and Alexandre Tkatchenko. Molecular force fields with gradient-domain machine learning: Construction and application to dynamics of small molecules with coupled cluster forces. *The Journal of Chemical Physics*, 150(11):114102, 2019. doi: 10.1063/1.5078687.

[50] Huziel E. Sauceda, Michael Gastegger, Stefan Chmiela, Klaus-Robert Müller, and Alexandre Tkatchenko. Molecular force fields with gradient-domain machine learning (gdml): Comparison and synergies with classical force fields, 2020.

[51] Hans W Horn, William C Swope, Jed W Pitera, Jeffry D Madura, Thomas J Dick, Greg L Hura, and Teresa Head-Gordon. Development of an improved four-site water model for biomolecular simulations: Tip4p-ew. *The Journal of chemical physics*, 120(20):9665–9678, 2004.

[52] Lee-Ping Wang, Keri A McKiernan, Joseph Gomes, Kyle A Beauchamp, Teresa Head-Gordon, Julia E Rice, William C Swope, Todd J Martínez, and Vijay S Pande. Building a more predictive protein force field: a systematic and reproducible route to amber-fb15. *The Journal of Physical Chemistry B*, 121(16):4023–4039, 2017.

[53] Eliot Boulanger, Lei Huang, Chetan Rupakheti, Alexander D MacKerell Jr, and Benoît Roux. Optimized lennard-jones parameters for druglike small molecules. *Journal of chemical theory and computation*, 14(6):3121–3131, 2018.

[54] SimonBoothroyd, Owen Madin, Jeff Wagner, Jeffry Setiadi, Jaime Rodríguez-Guerra, Matt Thompson, and David Dotson. openforcefield/openff-evaluator: 0.3.0, October 2020. URL https://doi.org/10.5281/zenodo.4153484.

[55] Justin S Smith, Olexandr Isayev, and Adrian E Roitberg. Ani-1: an extensible neural network potential with dft accuracy at force field computational cost. *Chemical science*, 8(4):3192–3203, 2017.

[56] Kristof Schütt, Pieter-Jan Kindermans, Huziel Enoc Sauceda Felix, Stefan Chmiela, Alexandre Tkatchenko, and Klaus-Robert Müller. Schnet: A continuous-filter convolutional neural network for modeling quantum interactions. In *Advances in neural information processing systems*, pages 991–1001, 2017.

[57] Kun Yao, John E. Herr, David W. Toth, Ryker Mckintyre, and John Parkhill. The tensormol-0.1 model chemistry: a neural network augmented with long-range physics. *Chem. Sci.*, 9:2261–2269, 2018. doi: 10.1039/C7SC04934J. URL http://dx.doi.org/10.1039/C7SC04934J.

[58] Matt J Harvey, Giovanni Giupponi, and G De Fabritiis. Acemd: accelerating biomolecular dynamics in the microsecond time scale. *Journal of chemical theory and computation*, 5(6):1632–1639, 2009.

[59] Romelia Salomon-Ferrer, Andreas W Gotz, Duncan Poole, Scott Le Grand, and Ross C Walker. Routine microsecond molecular dynamics simulations with amber on gpus. 2. explicit solvent particle mesh ewald. *Journal of chemical theory and computation*, 9(9):3878–3888, 2013.

[60] David Van Der Spoel, Erik Lindahl, Berk Hess, Gerrit Groenhof, Alan E Mark, and Herman JC Berendsen. Gromacs: fast, flexible, and free. *Journal of computational chemistry*, 26(16):1701–1718, 2005.

[61] Yue Wang, Yongbin Sun, Ziwei Liu, Sanjay E Sarma, Michael M Bronstein, and Justin M Solomon. Dynamic graph cnn for learning on point clouds. *Acm Transactions On Graphics (tog)*, 38(5):1–12, 2019.

[62] Jian Du, Shanghang Zhang, Guanhang Wu, Jose M. F. Moura, and Soummya Kar. Topology Adaptive Graph Convolutional Networks. *arXiv:1710.10370 [cs, stat]*, February 2018.

[63] Felix Wu, Tianyi Zhang, Amauri Holanda de Souza Jr, Christopher Fifty, Tao Yu, and Kilian Q Weinberger. Simplifying graph convolutional networks. *arXiv preprint arXiv:1902.07153*, 2019.

[64] D.A. Case, K. Belfon, I.Y. Ben-Shalom, S.R. Brozell, D.S. Cerutti, T.E. Cheatham, III, V.W.D. Cruzeiro, T.A. Darden, R.E. Duke, G. Giambasu, M.K. Gilson, H. Gohlke, R Harris A.W. Goetz, S. Izadi, S.A. Izmailov, K. Kasavajhala, A. Kovalenko, R. Krasny, T. Kurtzman, T.S. Lee, S. LeGrand, P. Li, C. Lin, J. Liu, T. Luchko, R. Luo, V. Man, K.M. Merz, Y. Miao, O. Mikhailovskii, G. Monard, H. Nguyen, A. Onufriev, F. Pan, S. Pantano, R. Qi, D.R. Roe, A. Roitberg, C. Sagui, S. Schott-Verdugo, J. Shen, C.L. Simmerling, N.R. Skrynnikov, J. Smith, J. Swails, R.C. Walker, J. Wang, L. Wilson, R.M. Wolf, X. Wu, Y. Xiong, Y. Xue, D.M. York, and P.A. Kollman. Amber 2020, 2020.

[65] Rodrigo Galindo-Murillo, James C Robertson, Marie Zgarbova, Jiri Sponer, Michal Otyepka, Petr Jurecka, and Thomas E Cheatham III. Assessing the current state of amber force field modifications for dna. *Journal of chemical theory and computation*, 12(8):4114–4127, 2016.

[66] Alberto Pérez, Iván Marchán, Daniel Svozil, Jiri Sponer, Thomas E Cheatham III, Charles A Laughton, and Modesto Orozco. Refinement of the amber force field for nucleic acids: improving the description of α/γ conformers. *Biophysical journal*, 92(11):3817–3829, 2007.

[67] In Suk Joung and Thomas E Cheatham III. Molecular dynamics simulations of the dynamic and energetic properties of alkali and halide ions using water-model-specific ion parameters. *The Journal of Physical Chemistry B*, 113(40): 13279–13290, 2009.

[68] Pengfei Li, Benjamin P Roberts, Dhruva K Chakravorty, and Kenneth M Merz Jr. Rational design of particle mesh ewald compatible lennard-jones parameters for+ 2 metal cations in explicit solvent. *Journal of chemical theory and computation*, 9(6):2733–2748, 2013.

[69] Pengfei Li and Kenneth M Merz Jr. Taking into account the ion-induced dipole interaction in the nonbonded model of ions. *Journal of chemical theory and computation*, 10(1):289–297, 2014.

[70] Pengfei Li, Lin Frank Song, and Kenneth M Merz Jr. Parameterization of highly charged metal ions using the 12-6-4 lj-type nonbonded model in explicit water. *The Journal of Physical Chemistry B*, 119(3):883–895, 2015.

[71] Callum J Dickson, Benjamin D Madej, Åge A Skjevik, Robin M Betz, Knut Teigen, Ian R Gould, and Ross C Walker. Lipid14: the amber lipid force field. *Journal of chemical theory and computation*, 10(2):865–879, 2014.

[72] Karl N Kirschner, Austin B Yongye, Sarah M Tschampel, Jorge González-Outeiriño, Charlisa R Daniels, B Lachele Foley, and Robert J Woods. Glycam06: a generalizable biomolecular force field. carbohydrates. *Journal of computational chemistry*, 29(4):622–655, 2008.

[73] Mari L DeMarco and Robert J Woods. Atomic-resolution conformational analysis of the gm3 ganglioside in a lipid bilayer and its implications for ganglioside–protein recognition at membrane surfaces. *Glycobiology*, 19(4):344–355, 2009.

[74] Mari L DeMarco, Robert J Woods, James H Prestegard, and Fang Tian. Presentation of membrane-anchored glycosphingolipids determined from molecular dynamics simulations and nmr paramagnetic relaxation rate enhancement. *Journal of the American Chemical Society*, 132(4):1334–1338, 2010.

[75] George A Khoury, Jeff P Thompson, James Smadbeck, Chris A Kieslich, and Christodoulos A Floudas. Forcefield_ptm: Ab initio charge and amber forcefield parameters for frequently occurring post-translational modifications. *Journal of chemical theory and computation*, 9(12):5653–5674, 2013.

[76] Dazhi Tan, Stefano Piana, Robert M Dirks, and David E Shaw. Rna force field with accuracy comparable to state-of-the-art protein force fields. *Proceedings of the National Academy of Sciences*, 115(7):E1346–E1355, 2018.

[77] Shama Khan, Imane Bjij, Fisayo A Olotu, Clement Agoni, Emmanuel Adeniji, and Mahmoud E S Soliman. Covalent simulations of covalent/irreversible enzyme inhibition in drug discovery: a reliable technical protocol. *Future medicinal chemistry*, 10(19):2265–2275, 2018.

[78] B Weisfeiler and A Leman. The reduction of a graph to canonical form and the algebgra which appears therein. 1968.

[79] Kurt Hornik. Approximation capabilities of multilayer feedforward networks. *Neural networks*, 4(2):251–257, 1991.

[80] Kurt Hornik, Maxwell Stinchcombe, Halbert White, et al. Multilayer feedforward networks are universal approximators. 1991.
